# (*E*)-3-{[([1,1′-Biphen­yl]-3-ylmeth­yl)iminium­yl]meth­yl}-6,8-di­chloro-2*H*-chromen-4-olate

**DOI:** 10.1107/S160053681302285X

**Published:** 2013-08-17

**Authors:** Yoshinobu Ishikawa, Yuya Motohashi

**Affiliations:** a School of Pharmaceutical Sciences, University of Shizuoka, 52-1 Yada, Suruga-ku, Shizuoka 422-8526, Japan

## Abstract

In the crystal of the title compound, C_23_H_17_Cl_2_NO_2_, the H atom of the –OH group is transferred to the N atom of the imine, forming a zwitterion. This results in a six-membered intra­molecular O⋯H—N hydrogen-bonded ring, rather than that formed with an O—H⋯N hydrogen bond. The dihedral angle between the rings of the biphenyl unit is 13.88 (10)°. In the crystal, mol­ecules are linked by N—H⋯O and C—H⋯O inter­actions.

## Related literature

For the biological propertries of similar structures, see: Khan *et al.* (2009 ▶); Tu *et al.* (2013 ▶). For related structures, see: Benaouida *et al.* (2013 ▶); Małecka & Budzisz (2006 ▶); Ishikawa & Motohashi (2013*a* ▶,*b* ▶).
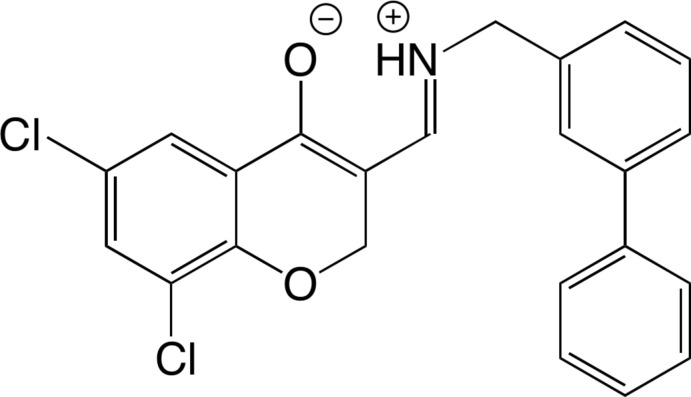


## Experimental

### 

#### Crystal data

C_23_H_17_Cl_2_NO_2_
*M* *_r_* = 410.30
Monoclinic, 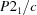

*a* = 17.996 (8) Å
*b* = 9.127 (6) Å
*c* = 11.649 (7) Å
β = 102.00 (4)°
*V* = 1871.5 (19) Å^3^
*Z* = 4
Mo *K*α radiation
μ = 0.37 mm^−1^
*T* = 100 K
0.40 × 0.25 × 0.25 mm

#### Data collection

Rigaku AFC-7R diffractometer
5086 measured reflections
4254 independent reflections
3499 reflections with *F* ^2^ > 2σ(*F* ^2^)
*R*_int_ = 0.090
3 standard reflections every 150 reflections intensity decay: −0.7%

#### Refinement

*R*[*F* ^2^ > 2σ(*F* ^2^)] = 0.045
*wR*(*F* ^2^) = 0.115
*S* = 1.01
4254 reflections
253 parameters
H-atom parameters constrained
Δρ_max_ = 0.58 e Å^−3^
Δρ_min_ = −0.69 e Å^−3^

### 

Data collection: *WinAFC* (Rigaku, 1999 ▶); cell refinement: *WinAFC*; data reduction: *WinAFC*; program(s) used to solve structure: *SHELXS97* (Sheldrick, 2008 ▶); program(s) used to refine structure: *SHELXL97* (Sheldrick, 2008 ▶); molecular graphics: *CrystalStructure* (Rigaku, 2010 ▶); software used to prepare material for publication: *CrystalStructure*.

## Supplementary Material

Crystal structure: contains datablock(s) General, I. DOI: 10.1107/S160053681302285X/hb7114sup1.cif

Structure factors: contains datablock(s) I. DOI: 10.1107/S160053681302285X/hb7114Isup2.hkl

Additional supplementary materials:  crystallographic information; 3D view; checkCIF report

## Figures and Tables

**Table 1:** Hydrogen-bond geometry (Å, °)

*D*—H⋯*A*	*D*—H	H⋯*A*	*D*⋯*A*	*D*—H⋯*A*
N5—H5⋯O4	0.88	2.20	2.811 (3)	126
N5—H5⋯O4^i^	0.88	2.38	3.081 (3)	137
C25—H25*A*⋯O4^ii^	0.99	2.59	3.546 (4)	164

Symmetry codes: (i) 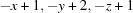; (ii) 
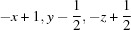
.

**Fig. 1.**
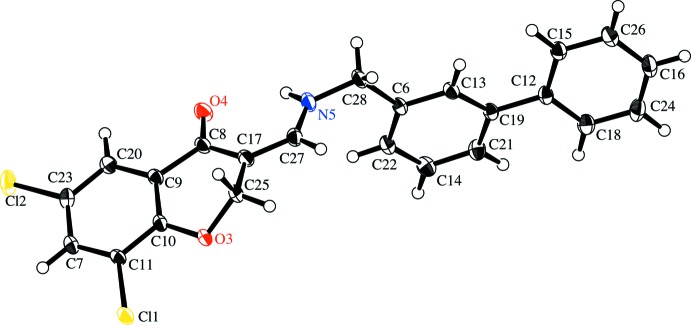
The molecular structure of the title compound, with displacement ellipsoids drawn at the 50% probability level.

## supplementary crystallographic information

### 1. Comment

Schiff bases of 3-formyl chromones have attracted much attention due to their biological functions such as enzyme inhibition (Khan *et al.* 2009; Tu *et al.* 2013). Here we report the crystal structure of the title compound, which was obtained from the condensation reaction of 6,8-dichloro-3-formylchromone with 3-phenylbenzylamine and successive reduction with 2-picoline borane. The structure shows that the H atom of the –OH group is transferred to the N5 atom of the imine, thus forming a zwitterion. As a result, an intramolecular O···H–N [O···N = 2.811 (3) Å], rather than O–H···N, hydrogen bond is formed. The bond distances O4–C8 [1.243 (3) Å], C8–C17 [1.426 (3) Å], C17–C27 [1.383 (3) Å] and C27–N5 [1.324 (3) Å] and torsion angles O4–C8–C17–C27 [–3.7 (3)°] and C8–C17–C27–N5 [1.2 (4)°] in the six-membered ring indicate charge delocalization among the atoms. This effect might be responsible for the preferential reduction of the α,β-unsaturated carbonyl of the synthetic intermediate, rather than reduction of the imine. The dihedral angle between the phenyl rings of the biphenyl group is 13.88 (10) °.

In the crystal, inversion dimers linked by pairs of N—H···O hydrogen bonds occur and the packing is consolidated by C—H···O interactions.

### 2. Experimental

3-Phenylbenzylamine (5.46 mmol), 6,8-dichloro-3-formylchromone (5.0 mmol) and 10 mg of *p*-toluenesulfonic acid were dissolved in 80 ml of benzene, and refluxed with Dean-Stark apparatus for 30 min. After cooling, the mixture was evaporated. To this residue, MeOH-AcOH (10:1, 60 ml) and 2-picoline borane (5 mmol) were added, and stirred overnight at room temperature. After the mixture was extracted with methylene chloride, the extract was washed with brine, dried over anhydrous Mg_2_SO_4_ and purified by column chromatography on silica gel (n-hexane: ethyl acetate = 6: 1). The eluted fractions were concentrated and filtered off. Layering n-hexane on the filtrate gave crystals (yield 23%), which were then recrystallized from ethyl acetate solution as yellow blocks.

### 3. Refinement

The carbon-bound hydrogen atoms were placed in geometrical positions [C–H 0.95 to 0.99 Å, *U*_iso_(H) = 1.2*U*_eq_(C)], and refined using a riding model. The hydrogen atom of the OH group was found to be located near N5 of the imine in a difference Fourier map, and refined with distance restraint [N–H 0.88 Å, *U*_iso_(H) = 1.2*U*_eq_(N)].

### Crystal data

**Table:**

C_23_H_17_Cl_2_NO_2_	*F*(000) = 848.00
*M**_r_* = 410.30	*D*_x_ = 1.456 Mg m^−^^3^
Monoclinic, *P*2_1_/*c*	Mo *K*α radiation, λ = 0.71069 Å
Hall symbol: -P 2ybc	Cell parameters from 25 reflections
*a* = 17.996 (8) Å	θ = 15.7–17.5°
*b* = 9.127 (6) Å	µ = 0.37 mm^−^^1^
*c* = 11.649 (7) Å	*T* = 100 K
β = 102.00 (4)°	Block, yellow
*V* = 1871.5 (19) Å^3^	0.40 × 0.25 × 0.25 mm
*Z* = 4	

### Data collection

**Table:**

Rigaku AFC-7R diffractometer	θ_max_ = 27.5°
ω–2θ scans	*h* = −13→23
5086 measured reflections	*k* = 0→11
4254 independent reflections	*l* = −15→14
3499 reflections with *F*^2^ > 2σ(*F*^2^)	3 standard reflections every 150 reflections
*R*_int_ = 0.090	intensity decay: −0.7%

### Refinement

**Table:**

Refinement on *F*^2^	Secondary atom site location: difference Fourier map
*R*[*F*^2^ > 2σ(*F*^2^)] = 0.045	Hydrogen site location: inferred from neighbouring sites
*wR*(*F*^2^) = 0.115	H-atom parameters constrained
*S* = 1.01	*w* = 1/[σ^2^(*F*_o_^2^) + (0.0505*P*)^2^ + 2.0486*P*] where *P* = (*F*_o_^2^ + 2*F*_c_^2^)/3
4254 reflections	(Δ/σ)_max_ < 0.001
253 parameters	Δρ_max_ = 0.58 e Å^−^^3^
0 restraints	Δρ_min_ = −0.69 e Å^−^^3^
Primary atom site location: structure-invariant direct methods	

### Special details

**Table:**

Refinement. Refinement was performed using all reflections. The weighted *R*-factor (*wR*) and goodness of fit (*S*) are based on *F*^2^. *R*-factor (gt) are based on *F*. The threshold expression of *F*^2^ > 2.0 σ(*F*^2^) is used only for calculating *R*-factor (gt).

### Fractional atomic coordinates and isotropic or equivalent isotropic displacement parameters (Å^2^)

**Table:**

	*x*	*y*	*z*	*U*_iso_*/*U*_eq_	
Cl1	0.80025 (2)	0.62806 (6)	0.19342 (4)	0.02502 (13)	
Cl2	0.86878 (3)	1.10381 (7)	0.46874 (5)	0.03284 (15)	
O3	0.64501 (7)	0.69744 (16)	0.20224 (12)	0.0213 (3)	
O4	0.57129 (8)	0.98666 (17)	0.42256 (13)	0.0251 (3)	
N5	0.42765 (9)	0.8598 (2)	0.34156 (15)	0.0228 (4)	
C6	0.32275 (10)	0.7096 (3)	0.39377 (16)	0.0189 (4)	
C7	0.82564 (10)	0.8699 (3)	0.32979 (16)	0.0193 (4)	
C8	0.59004 (10)	0.9131 (3)	0.34336 (17)	0.0203 (4)	
C9	0.67152 (10)	0.9075 (2)	0.33128 (16)	0.0181 (4)	
C10	0.69432 (10)	0.7945 (2)	0.26495 (15)	0.0174 (4)	
C11	0.77212 (10)	0.7748 (2)	0.26781 (15)	0.0177 (4)	
C12	0.13573 (10)	0.5180 (3)	0.40462 (15)	0.0188 (4)	
C13	0.24573 (10)	0.6722 (3)	0.37163 (16)	0.0192 (4)	
C14	0.34775 (11)	0.5149 (3)	0.53488 (17)	0.0244 (4)	
C15	0.08092 (10)	0.6104 (3)	0.33894 (17)	0.0216 (4)	
C16	−0.01848 (11)	0.4374 (3)	0.35077 (18)	0.0271 (5)	
C17	0.53989 (10)	0.8232 (3)	0.26182 (17)	0.0206 (4)	
C18	0.11133 (11)	0.3843 (3)	0.44211 (17)	0.0240 (4)	
C19	0.21812 (10)	0.5583 (2)	0.43059 (15)	0.0174 (4)	
C20	0.72538 (10)	1.0033 (3)	0.39433 (16)	0.0199 (4)	
C21	0.27091 (11)	0.4809 (3)	0.51376 (17)	0.0234 (4)	
C22	0.37403 (10)	0.6290 (3)	0.47505 (16)	0.0211 (4)	
C23	0.80161 (10)	0.9837 (3)	0.39174 (16)	0.0209 (4)	
C24	0.03495 (12)	0.3446 (3)	0.41568 (18)	0.0278 (5)	
C25	0.56977 (10)	0.7595 (3)	0.16214 (17)	0.0214 (4)	
C26	0.00452 (11)	0.5703 (3)	0.31196 (18)	0.0254 (4)	
C27	0.46441 (10)	0.8011 (3)	0.26553 (17)	0.0213 (4)	
C28	0.34598 (10)	0.8407 (3)	0.32960 (19)	0.0245 (4)	
H5	0.4531	0.9115	0.4006	0.0273*	
H7	0.8781	0.8573	0.3298	0.0231*	
H13	0.2111	0.7263	0.3145	0.0231*	
H14	0.3827	0.4597	0.5908	0.0293*	
H15	0.0960	0.7019	0.3123	0.0259*	
H16	−0.0706	0.4104	0.3329	0.0326*	
H18	0.1475	0.3194	0.4864	0.0288*	
H20	0.7101	1.0810	0.4385	0.0239*	
H21	0.2539	0.4036	0.5565	0.0280*	
H22	0.4267	0.6517	0.4897	0.0253*	
H24	0.0195	0.2534	0.4423	0.0334*	
H25A	0.5347	0.6821	0.1236	0.0256*	
H25B	0.5720	0.8369	0.1034	0.0256*	
H26	−0.0319	0.6342	0.2668	0.0305*	
H27	0.4362	0.7376	0.2079	0.0255*	
H28A	0.3226	0.8312	0.2452	0.0294*	
H28B	0.3250	0.9302	0.3589	0.0294*	

### Atomic displacement parameters (Å^2^)

**Table:**

	*U*^11^	*U*^22^	*U*^33^	*U*^12^	*U*^13^	*U*^23^
Cl1	0.0160 (2)	0.0271 (3)	0.0341 (3)	−0.00033 (17)	0.01027 (17)	−0.00751 (19)
Cl2	0.0235 (3)	0.0419 (4)	0.0342 (3)	−0.0135 (2)	0.00837 (19)	−0.0155 (3)
O3	0.0121 (6)	0.0221 (7)	0.0293 (7)	−0.0002 (5)	0.0033 (5)	−0.0028 (6)
O4	0.0202 (7)	0.0294 (8)	0.0273 (7)	0.0023 (6)	0.0089 (6)	−0.0024 (6)
N5	0.0145 (7)	0.0272 (9)	0.0273 (8)	−0.0018 (7)	0.0056 (6)	0.0010 (7)
C6	0.0147 (8)	0.0237 (10)	0.0196 (8)	−0.0003 (7)	0.0067 (7)	−0.0000 (7)
C7	0.0139 (8)	0.0257 (10)	0.0186 (8)	−0.0009 (7)	0.0043 (7)	0.0031 (7)
C8	0.0158 (8)	0.0216 (9)	0.0253 (9)	0.0025 (7)	0.0084 (7)	0.0053 (8)
C9	0.0153 (8)	0.0213 (9)	0.0188 (8)	0.0012 (7)	0.0058 (7)	0.0042 (7)
C10	0.0132 (8)	0.0208 (9)	0.0184 (8)	−0.0003 (7)	0.0036 (6)	0.0038 (7)
C11	0.0146 (8)	0.0213 (9)	0.0186 (8)	0.0025 (7)	0.0069 (7)	0.0016 (7)
C12	0.0170 (8)	0.0246 (10)	0.0160 (8)	−0.0036 (7)	0.0061 (7)	−0.0036 (7)
C13	0.0156 (8)	0.0226 (10)	0.0204 (9)	0.0011 (7)	0.0058 (7)	0.0017 (7)
C14	0.0211 (9)	0.0314 (11)	0.0195 (9)	0.0023 (8)	0.0015 (7)	0.0035 (8)
C15	0.0191 (9)	0.0237 (10)	0.0230 (9)	−0.0020 (8)	0.0066 (7)	−0.0003 (8)
C16	0.0188 (9)	0.0342 (12)	0.0302 (10)	−0.0083 (8)	0.0090 (8)	−0.0097 (9)
C17	0.0149 (8)	0.0227 (10)	0.0247 (9)	0.0019 (7)	0.0050 (7)	0.0045 (8)
C18	0.0230 (9)	0.0276 (11)	0.0222 (9)	−0.0031 (8)	0.0066 (7)	0.0004 (8)
C19	0.0158 (8)	0.0219 (9)	0.0156 (8)	−0.0009 (7)	0.0058 (7)	−0.0021 (7)
C20	0.0202 (9)	0.0229 (9)	0.0178 (8)	0.0001 (8)	0.0068 (7)	0.0002 (7)
C21	0.0231 (9)	0.0276 (11)	0.0202 (9)	−0.0036 (8)	0.0061 (7)	0.0043 (8)
C22	0.0148 (8)	0.0273 (10)	0.0210 (9)	−0.0014 (8)	0.0035 (7)	−0.0029 (8)
C23	0.0188 (9)	0.0260 (10)	0.0180 (8)	−0.0064 (8)	0.0041 (7)	0.0000 (7)
C24	0.0279 (11)	0.0287 (11)	0.0296 (10)	−0.0113 (9)	0.0122 (8)	−0.0035 (9)
C25	0.0126 (8)	0.0242 (10)	0.0264 (9)	−0.0002 (7)	0.0019 (7)	0.0004 (8)
C26	0.0172 (9)	0.0317 (11)	0.0276 (10)	−0.0009 (8)	0.0050 (8)	−0.0027 (9)
C27	0.0171 (9)	0.0222 (10)	0.0243 (9)	−0.0014 (7)	0.0037 (7)	0.0048 (8)
C28	0.0128 (8)	0.0277 (11)	0.0338 (11)	0.0017 (8)	0.0069 (7)	0.0087 (9)

### Geometric parameters (Å, º)

**Table:**

Cl1—C11	1.727 (3)	C16—C24	1.383 (3)
Cl2—C23	1.737 (2)	C16—C26	1.387 (4)
O3—C10	1.355 (3)	C17—C25	1.495 (3)
O3—C25	1.452 (3)	C17—C27	1.383 (3)
O4—C8	1.243 (3)	C18—C24	1.393 (3)
N5—C27	1.324 (3)	C19—C21	1.399 (3)
N5—C28	1.458 (3)	C20—C23	1.390 (3)
C6—C13	1.398 (3)	N5—H5	0.880
C6—C22	1.388 (3)	C7—H7	0.950
C6—C28	1.515 (3)	C13—H13	0.950
C7—C11	1.383 (3)	C14—H14	0.950
C7—C23	1.384 (3)	C15—H15	0.950
C8—C9	1.503 (3)	C16—H16	0.950
C8—C17	1.426 (3)	C18—H18	0.950
C9—C10	1.400 (3)	C20—H20	0.950
C9—C20	1.395 (3)	C21—H21	0.950
C10—C11	1.405 (3)	C22—H22	0.950
C12—C15	1.398 (3)	C24—H24	0.950
C12—C18	1.397 (3)	C25—H25A	0.990
C12—C19	1.496 (3)	C25—H25B	0.990
C13—C19	1.393 (3)	C26—H26	0.950
C14—C21	1.388 (3)	C27—H27	0.950
C14—C22	1.390 (3)	C28—H28A	0.990
C15—C26	1.394 (3)	C28—H28B	0.990
			
C10—O3—C25	112.38 (16)	C15—C26—C16	120.20 (19)
C27—N5—C28	121.46 (17)	N5—C27—C17	126.35 (18)
C13—C6—C22	119.33 (19)	N5—C28—C6	115.05 (16)
C13—C6—C28	117.78 (16)	C27—N5—H5	119.284
C22—C6—C28	122.86 (17)	C28—N5—H5	119.257
C11—C7—C23	118.94 (18)	C11—C7—H7	120.532
O4—C8—C9	120.57 (17)	C23—C7—H7	120.528
O4—C8—C17	124.98 (18)	C6—C13—H13	118.915
C9—C8—C17	114.38 (18)	C19—C13—H13	118.918
C8—C9—C10	118.70 (17)	C21—C14—H14	119.697
C8—C9—C20	120.93 (18)	C22—C14—H14	119.698
C10—C9—C20	120.04 (18)	C12—C15—H15	119.481
O3—C10—C9	123.01 (17)	C26—C15—H15	119.484
O3—C10—C11	117.81 (17)	C24—C16—H16	120.227
C9—C10—C11	119.09 (16)	C26—C16—H16	120.219
Cl1—C11—C7	120.24 (15)	C12—C18—H18	119.399
Cl1—C11—C10	118.81 (14)	C24—C18—H18	119.395
C7—C11—C10	120.95 (18)	C9—C20—H20	120.358
C15—C12—C18	117.77 (17)	C23—C20—H20	120.373
C15—C12—C19	121.26 (18)	C14—C21—H21	119.425
C18—C12—C19	120.95 (17)	C19—C21—H21	119.422
C6—C13—C19	122.17 (17)	C6—C22—H22	120.266
C21—C14—C22	120.61 (18)	C14—C22—H22	120.267
C12—C15—C26	121.0 (2)	C16—C24—H24	119.886
C24—C16—C26	119.55 (19)	C18—C24—H24	119.877
C8—C17—C25	117.46 (17)	O3—C25—H25A	109.370
C8—C17—C27	123.6 (2)	O3—C25—H25B	109.364
C25—C17—C27	118.80 (17)	C17—C25—H25A	109.360
C12—C18—C24	121.21 (19)	C17—C25—H25B	109.359
C12—C19—C13	121.59 (16)	H25A—C25—H25B	107.995
C12—C19—C21	121.14 (18)	C15—C26—H26	119.894
C13—C19—C21	117.25 (17)	C16—C26—H26	119.907
C9—C20—C23	119.27 (19)	N5—C27—H27	116.830
C14—C21—C19	121.2 (2)	C17—C27—H27	116.823
C6—C22—C14	119.47 (18)	N5—C28—H28A	108.504
Cl2—C23—C7	118.91 (15)	N5—C28—H28B	108.510
Cl2—C23—C20	119.46 (16)	C6—C28—H28A	108.504
C7—C23—C20	121.62 (18)	C6—C28—H28B	108.506
C16—C24—C18	120.2 (3)	H28A—C28—H28B	107.520
O3—C25—C17	111.33 (15)		
			
C10—O3—C25—C17	−53.6 (2)	C15—C12—C18—H18	179.6
C10—O3—C25—H25A	−174.6	C18—C12—C15—C26	0.1 (3)
C10—O3—C25—H25B	67.3	C18—C12—C15—H15	−179.9
C25—O3—C10—C9	29.1 (3)	C15—C12—C19—C13	−13.5 (3)
C25—O3—C10—C11	−154.42 (15)	C15—C12—C19—C21	167.82 (17)
C27—N5—C28—C6	91.3 (3)	C19—C12—C15—C26	178.03 (16)
C27—N5—C28—H28A	−30.5	C19—C12—C15—H15	−2.0
C27—N5—C28—H28B	−147.0	C18—C12—C19—C13	164.43 (17)
C28—N5—C27—C17	173.51 (17)	C18—C12—C19—C21	−14.3 (3)
C28—N5—C27—H27	−6.5	C19—C12—C18—C24	−178.38 (16)
H5—N5—C27—C17	−6.5	C19—C12—C18—H18	1.6
H5—N5—C27—H27	173.5	C6—C13—C19—C12	−179.00 (16)
H5—N5—C28—C6	−88.7	C6—C13—C19—C21	−0.3 (3)
H5—N5—C28—H28A	149.5	H13—C13—C19—C12	1.0
H5—N5—C28—H28B	33.0	H13—C13—C19—C21	179.7
C13—C6—C22—C14	−1.5 (3)	C21—C14—C22—C6	0.2 (3)
C13—C6—C22—H22	178.6	C21—C14—C22—H22	−179.8
C22—C6—C13—C19	1.5 (3)	C22—C14—C21—C19	1.1 (3)
C22—C6—C13—H13	−178.5	C22—C14—C21—H21	−178.9
C13—C6—C28—N5	−170.51 (17)	H14—C14—C21—C19	−178.9
C13—C6—C28—H28A	−48.8	H14—C14—C21—H21	1.1
C13—C6—C28—H28B	67.8	H14—C14—C22—C6	−179.8
C28—C6—C13—C19	−176.64 (17)	H14—C14—C22—H22	0.2
C28—C6—C13—H13	3.4	C12—C15—C26—C16	0.4 (3)
C22—C6—C28—N5	11.4 (3)	C12—C15—C26—H26	−179.6
C22—C6—C28—H28A	133.1	H15—C15—C26—C16	−179.6
C22—C6—C28—H28B	−110.3	H15—C15—C26—H26	0.4
C28—C6—C22—C14	176.60 (17)	C24—C16—C26—C15	−0.4 (4)
C28—C6—C22—H22	−3.4	C24—C16—C26—H26	179.5
C11—C7—C23—Cl2	−179.57 (15)	C26—C16—C24—C18	0.1 (4)
C11—C7—C23—C20	0.9 (3)	C26—C16—C24—H24	−179.9
C23—C7—C11—Cl1	−177.89 (15)	H16—C16—C24—C18	−179.9
C23—C7—C11—C10	1.5 (3)	H16—C16—C24—H24	0.1
H7—C7—C11—Cl1	2.1	H16—C16—C26—C15	179.6
H7—C7—C11—C10	−178.5	H16—C16—C26—H26	−0.5
H7—C7—C23—Cl2	0.4	C8—C17—C25—O3	45.8 (3)
H7—C7—C23—C20	−179.1	C8—C17—C25—H25A	166.8
O4—C8—C9—C10	162.18 (17)	C8—C17—C25—H25B	−75.2
O4—C8—C9—C20	−11.3 (3)	C8—C17—C27—N5	1.2 (4)
O4—C8—C17—C25	171.54 (17)	C8—C17—C27—H27	−178.8
O4—C8—C17—C27	−3.7 (3)	C25—C17—C27—N5	−173.96 (17)
C9—C8—C17—C25	−11.6 (3)	C25—C17—C27—H27	6.0
C9—C8—C17—C27	173.26 (16)	C27—C17—C25—O3	−138.79 (17)
C17—C8—C9—C10	−14.9 (3)	C27—C17—C25—H25A	−17.8
C17—C8—C9—C20	171.67 (16)	C27—C17—C25—H25B	100.2
C8—C9—C10—O3	6.2 (3)	C12—C18—C24—C16	0.3 (3)
C8—C9—C10—C11	−170.28 (15)	C12—C18—C24—H24	−179.7
C8—C9—C20—C23	172.43 (15)	H18—C18—C24—C16	−179.7
C8—C9—C20—H20	−7.6	H18—C18—C24—H24	0.3
C10—C9—C20—C23	−0.9 (3)	C12—C19—C21—C14	177.69 (16)
C10—C9—C20—H20	179.1	C12—C19—C21—H21	−2.3
C20—C9—C10—O3	179.67 (16)	C13—C19—C21—C14	−1.1 (3)
C20—C9—C10—C11	3.2 (3)	C13—C19—C21—H21	178.9
O3—C10—C11—Cl1	−0.8 (3)	C9—C20—C23—Cl2	179.29 (15)
O3—C10—C11—C7	179.83 (14)	C9—C20—C23—C7	−1.2 (3)
C9—C10—C11—Cl1	175.86 (14)	H20—C20—C23—Cl2	−0.7
C9—C10—C11—C7	−3.5 (3)	H20—C20—C23—C7	178.8
C15—C12—C18—C24	−0.4 (3)		

### Hydrogen-bond geometry (Å, º)

**Table:**

*D*—H···*A*	*D*—H	H···*A*	*D*···*A*	*D*—H···*A*
N5—H5···O4	0.88	2.20	2.811 (3)	126
N5—H5···O4^i^	0.88	2.38	3.081 (3)	137
C25—H25*A*···O4^ii^	0.99	2.59	3.546 (4)	164

Symmetry codes: (i) −*x*+1, −*y*+2, −*z*+1; (ii) −*x*+1, *y*−1/2, −*z*+1/2.
